# The clinicopathological significance of thymic epithelial markers expression in thymoma and thymic carcinoma

**DOI:** 10.1186/s12885-023-10619-6

**Published:** 2023-02-16

**Authors:** Huiyang Li, Bo Ren, Shili Yu, Hongwen Gao, Ping-Li Sun

**Affiliations:** Department of Pathology, The Second Hospital of Jilin University, 218 Ziqiang Road, 130041 Changchun, Jilin China

**Keywords:** Immunohistochemical staining, Thymic epithelial markers, Cortex, Medulla, Differentiation, Thymoma, Thymic squamous cell carcinoma

## Abstract

**Background:**

The classification of thymomas is based on the morphology of epithelial tumor cells and the proportion of lymphocytes. Type A thymomas are composed of the spindle or oval tumor epithelial cells. Tumor cells of B thymomas are epithelioid-shaped with increasing atypia. Type AB thymomas have the features of epithelial tumor cells of A and B thymomas. The diagnosis can be difficult because of the complex morphology. Some novel thymic epithelial markers have been reported in several preclinical studies, but they have not been applied to clinical practice. Here, we investigated the expression of 3 cortical and 3 medullary markers, which are thymoproteasome-specific subunit β5t (β5t), thymus-specific serine protease 16 (PRSS16), cathepsin V, autoimmune regulator (AIRE), CD40 and claudin-4.

**Methods:**

Immunohistochemistry was used to analyze 53 cases of thymomas and thymic squamous cell carcinomas (TSCC), aiming to explore the expression of cortical and medullary epithelial markers and their correlation with histological classification, Masaoka-Koga stage, and prognosis.

**Results:**

Our results found that for cortical epithelial markers the expression of β5t, PRSS16, and cathepsin V was higher in type AB and B thymomas than in micronodular thymoma with lymphoid stroma (MNT), and we observed a dramatic increase of β5t and PRSS16 expression in type AB compared to type A thymomas. In medullary epithelial markers, the expression of AIRE was higher in type A than in B3 thymomas. CD40 and β5t expression were associated with the Masaoka-Koga stage. High cathepsin V expression was related to a good prognosis and a longer progression-free survival.

**Conclusion:**

This is the first comprehensive analysis of the role of thymic cortical and medullary epithelial markers as biomarkers for differential diagnosis and prognosis in thymic tumors. Thymic medullary epithelial immunophenotype was found to exhibit in type A, MNT, and TSCC. Type B thymomas primarily exhibited a cortical epithelial immunophenotype. Type AB thymomas showed cortical, medullary, or mixed corticomedullary epithelial immunophenotype. Our results demonstrated that thymic cortical and medullary epithelial markers including β5t, PRSS16, cathepsin V, and AIRE could be used as ancillary markers in the diagnosis and prognosis of thymic epithelial tumors.

## Background

Thymic epithelial tumors (TETs) are rare tumors, including thymoma, thymic carcinoma, and thymic neuroendocrine tumors. Thymoma represents the most common TETs, with an annual incidence rate of 0.13–0.26 per 100,000 people [1].

The histopathologic classification of TETs is a controversial issue, and 24 histologic classifications of thymoma have been introduced in the past [2]. A previous study has divided thymoma into lymphocyte-predominant, epithelial-predominant, mixed (lymphoepithelial), and spindle cell thymoma [3]. In 1985, Marino and Muller-Hermelink divided thymic epithelial tumors into medullary, mixed medullary and cortical, predominantly cortical, and cortical thymoma, well-differentiated thymic carcinoma, and high-grade carcinoma [4]. Currently, the most widely used classification system for TETs is World Health Organization (WHO) classification. According to the atypia of the epithelial cells and the extent of the lymphocyte component, TETs are subclassified into type A, AB, B1, B2, B3 thymoma, metaplastic thymoma, micronodular thymoma with lymphoid stroma (MNT), thymic carcinoma, and thymic neuroendocrine tumors in the fifth edition of the WHO classification of thymic epithelial tumors [1]. Many reports have investigated the clinicopathologic relationship between WHO histologic classification of thymoma and prognosis. The WHO histological classification was shown to correlate with disease-free survival and recurrence by some studies [5–7], but the relationship between WHO classification and overall survival (OS) remains unclear [8, 9]. Moreover, because of the rarity and heterogeneous behaviors of thymomas, there have been arguments and controversies regarding the reproducibility of WHO classification [10–12].

It is difficult for pathologists to distinguish subtypes of TETs through histological features of epithelial cells and lymphocytes in some cases. Researchers have explored some markers to differentiate thymoma from thymic carcinoma, such as CD5, CD117, CD70, thymoproteasome-specific subunit β5t (β5t), Glucose transporter 1 (Glut-1), BRCA1 associated protein 1, methylthioadenosine phosphorylase, Terminal deoxynucleotidyl transferase (TdT) and Ki-67 labeling index [13–16]. However, no one single biomarker can distinguish different thymomas effectively, hence a mix of antibodies is frequently used to aid the diagnosis. In thymoma, lymphocytes can be identified by CD3, CD5, CD20, CD1α, and TdT. Although CD20 can be expressed by some type A and AB thymoma epithelial cells, the generally used epithelial markers CK, CK5/6, p40, and p63 have limited utility in thymoma histological classification [17]. The fifth edition of the “WHO Classification of Thoracic Tumors” mentioned three thymic cortical and three medullary epithelial markers, which are β5t, Thymus-specific serine protease 16 (PRSS16), cathepsin V, autoimmune regulator (AIRE), CD40 and claudin-4, but no indication was given about their roles in histological classification [1]. Previous studies mainly focused on biological functions. β5t was mainly expressed in type B, and AB thymomas, and the expression of β5t correlated with the morphology of tumor cells and the number of TdT-positive lymphocytes [18–21]. Cathepsin V is mainly expressed in cortical thymic epithelial cells (cTECs), and its expression is increased in thymoma patients with myasthenia gravis [22]. PRSS16 is specifically expressed in the cTECs, correlates with the presentations of the self-peptides that are bound to MHC class II molecules, and is involved in the positive selection of CD4^+^ thymocytes [23]. AIRE expression is inherent to all medullary thymic epithelial cells (mTECs), and it can be expressed in different phases of differentiation [24]. In the final stage of maturation, mTECs lose AIRE expression accompanied by the formation of Hassall’s corpuscles [25]. CD40 is expressed in both mTECs and cTECs, mainly in mTECs, but Hassall’s corpuscles are stained very weakly [26]. Claudin-4 is found in the area surrounding Hassall’s corpuscles [27]. However, the role of thymic cortical and medullary epithelial markers in histological classification, Masaoka-Koga stage, and prognosis has hardly been studied so far.

In this study, we aimed to explore the expression of thymic cortical epithelial markers (β5t, PRSS16, and cathepsin V) and medullary epithelial markers (claudin-4, CD40, and AIRE) in thymoma and TSCC and its correlation with histological classification, staging, and prognosis.

## Methods

### Patients and specimens

We enrolled 53 patients diagnosed with thymoma or TSCC in the Department of Pathology, Second Hospital of Jilin University from December 2011 to May 2021, including 5 patients with type A thymoma, 5 with B1, 13 with B2, 5 with B3, 13 with AB, 5 with MNT, and 7 with TSCC, and all type A thymomas were classic and excluded atypical A thymoma. Tumor classification was based on the fifth WHO classification of Thoracic Tumors, and staging was performed using the Masaoka-Koga staging system. Follow-up data were recorded for 34 of 53 thymomas and TSCC. The mean follow-up period was 35.5 months (range 2–75 months). Among them, 3 patients died, 2 developed recurrences 2 and 45 months after the initial resection, and 1 showed metastasis to the right pleura 15 months postoperatively.

### Immunohistochemical staining

Formalin-fixed paraffin sections were deparaffinized in xylene, rehydrated in graded alcohol, and incubated in a 0.3% hydrogen peroxide solution for 30 min at room temperature to block endogenous peroxidase activity. For immunohistochemical analysis, the antibodies employed included β5t (rabbit polyclonal, 1:200, absin), PRSS16 (rabbit polyclonal, 1:500, cusabio), cathepsin V (rabbit polyclonal, 1:200, cusabio), claudin-4 (1:300, absin), CD40 (rabbit polyclonal, 1:300, cusabio), and AIRE (rabbit polyclonal, 1:200, absin). All procedures were performed according to the manufacturer’s instructions. The negative controls underwent the same procedures, and the primary antibody was replaced with PBS. Peritumor remnant thymus served as a positive control.

A semi-quantitative score was obtained by multiplying the grades for the extent (the percentage of positive tumor cells: 0–100%) and intensity (0 = negative, 1 = weak, 2 = intermediate, and 3 = strong) of the staining. The median was used as the cut-off value for distinguishing positive or negative. The quantity of positive cortical or medullary markers determines the immunophenotype of epithelial cells. Cortical epithelial immunophenotype was defined when the expression number of cortical antibodies exceeds the number of medullary antibodies and vice versa. It was thought to have a bidirectional corticomedullary immunophenotype when the number was equal. The case was defined as null immunophenotype if neither cortical nor medullary epithelial markers are expressed.

### Statistical analysis

All statistical analyses were performed using SPSS 26.0 (SPSS Inc, Chicago, IL). The results of the immunohistochemical analysis were evaluated by the Chi-square test or Fisher exact test. OS rates and progression-free survival (PFS) were calculated using the Kaplan-Meier method, and statistical significance was assessed by the log-rank test. The correlation between the protein expression and histological classification was analyzed using Spearman’s correlation. A Venn illustration was used to show corticomedullary epithelial immunophenotype between type A, B, and AB thymomas [28]. All *p* values were based on two-sided statistical analysis, and *p* values less than 0.05 were considered significant.

## Results

### Expression of cortical epithelial markers (β5t, PRSS16, and cathepsin V) in thymoma and TSCC

**Fig. 1:**
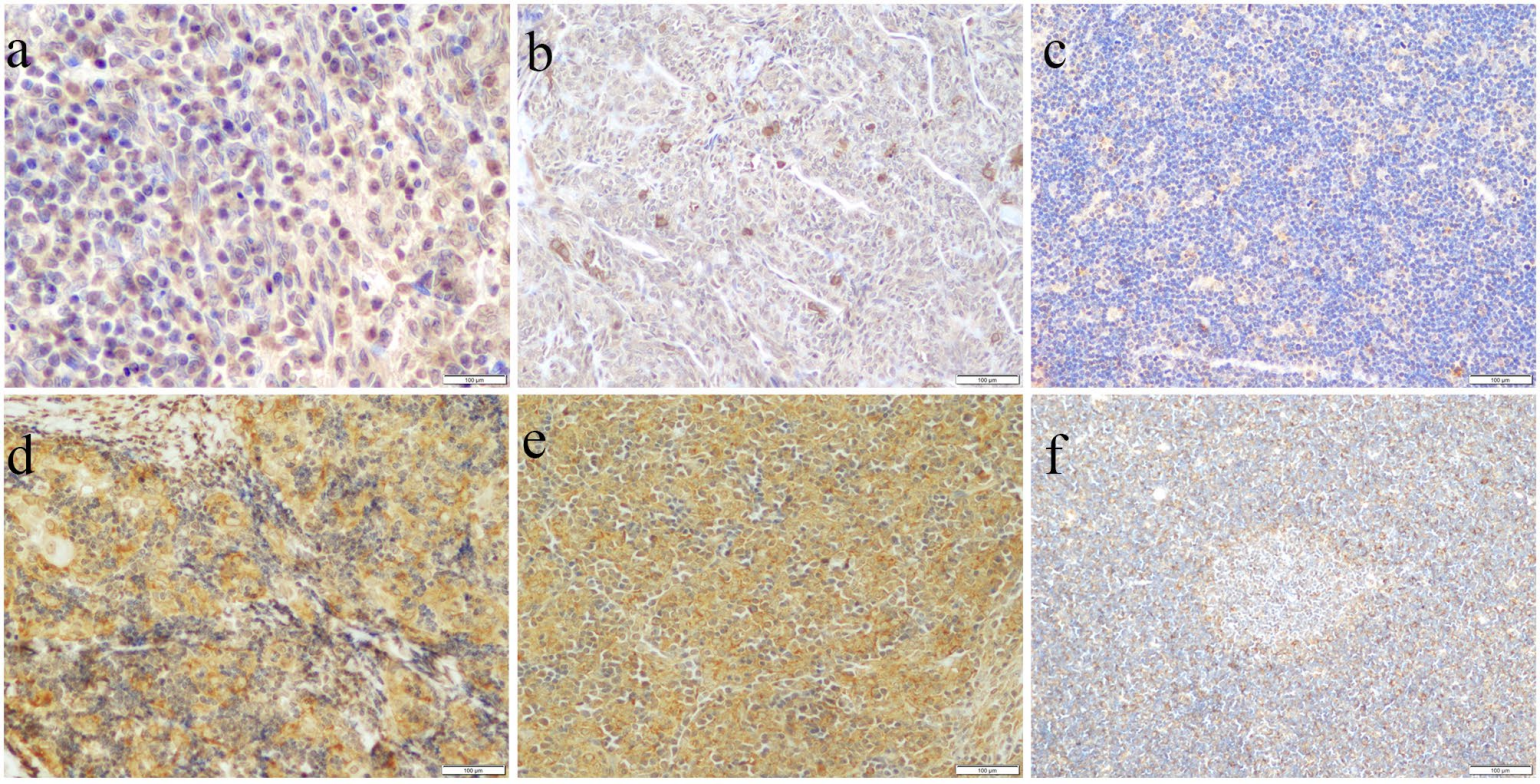
Expression of cortical epithelial markers in thymoma and TSCC. (a) β5t in AB thymoma × 400; (b) β5t in A thymoma × 200; (c) PRSS16 in B1 thymoma × 200; (d) PRSS16 in B2 thymoma × 200; (e) Cathepsin V in AB thymoma × 200; (f) Cathepsin V in B1 thymoma × 100

We stained 53 cases of thymoma and TSCC with 3 cortical markers (Table 1). β5t positivity was observed in the tumor cell nuclear in 76.9% (10/13) of type AB thymomas (Fig. 1A). In a few cases of AB thymomas with intricately intermingled type A component and type B-like component, positive β5t expression was detected in oval epithelial cells rather than spindle cells as in type A thymomas (Fig. 1A). β5t was positive in most type B thymomas (65.2%, 15/23), and its expression increased sequentially in B1-B3 thymomas. One case in type A and one case in TSCC was β5t positive, and β5t expression was observed in the plasma of tumor cells of type A thymoma (Fig. 1B).

**Table 1:** Expression of cortical and medullary markers in thymomas and TSCC

Classification	Cortical markers (%)			Medullary markers (%)		
	β5t	PRSS16	Cathepsin V	Claudin-4	CD40	AIRE
MNT	0	0	0	2(40.0)	2(40.0)	2(40.0)
A	1(20.0)	0	2(40.0)	1(20.0)	3(60.0)	5(100.0)
AB	10(76.9)	8(61.5)	9(69.2)	9(69.2)	8(61.5)	10(76.9)
B1	2(40.0)	4(80.0)	2(40.0)	1(20.0)	4(80.0)	4(80.0)
B2	9(69.2)	11(84.6)	10(76.9)	7(53.8)	8(61.5)	7(53.8)
B3	4(80.0)	3(60.0)	4(80.0)	2(40.0)	1(20.0)	0
TSCC	1(14.3)	2(28.6)	2(28.6)	6(85.7)	4(57.1)	2(28.6)

PRSS16 was expressed in 78.3% (18/23) of type B, and 61.5% (8/13) of type AB thymoma, not in MNT and type A thymoma, and rarely expressed in TSCC (28.6%, 2/7). Among type B thymomas, B2 thymoma (84.6%, 11/13) had the highest expression rate, followed by B1(80.0%, 4/5), and B3(60.0%, 3/5). PRSS16-positive epithelial cells were scattered in B1 thymoma (Fig. 1C), and a network of PRSS16-positive epithelial cells was seen in B2 (Fig. 1D) and B3 thymoma, which was consistent with the morphology of type B thymomas.

Cathepsin V expression was found in 69.6% (16/23) of type B, 69.2% (9/13) of type AB (Fig. 1E), 40.0% (2/5) of type A thymomas, and 28.6% (2/7) of TSCC, while MNT was not expressed. In type B thymoma, the expression of B2 (76.9%, 10/13) and B3 (80.0%, 4/5) thymoma was similar. The expression of cathepsin V in the medullary islands of B1 (Fig. 1F) was weaker than that in the cortex-like areas. Table 2 summarized the expression of cortical epithelial markers in the differential diagnosis. The expression of β5t, PRSS16, and cathepsin V was higher in type AB and B thymomas than in MNT (p < 0.05). Moreover, the expression of β5t and PRSS16 has significantly increased in type AB thymomas as compared with type A thymomas (p < 0.05). No significant differences were found in the rest groups (p > 0.05).

**Table 2:** Comparison of cortical and medullary markers expression in thymomas and TSCC

Classification	P value^*^					
	β5t	PRSS16	Cathepsin V	Claudin-4	CD40	AIRE
AB vs. MNT	0.007	0.036	0.029	0.326	0.608	0.268
B vs. MNT	0.013	0.003	0.008	1.000	0.639	1.000
AB vs. B1	0.268	0.615	0.326	0.118	0.615	1.000
AB vs. B2	1.000	0.378	1.000	0.688	1.000	0.411
AB vs. B3	1.000	1.000	1.000	0.326	0.294	0.007
A vs. B3	0.206	0.167	0.524	0.500	0.524	0.008
A vs. AB	0.047	0.036	0.326	0.118	1.000	0.522
B3 vs. TSCC	0.072	0.558	0.242	0.222	0.293	0.470

^*^ Fisher exact test

### Expression of medullary epithelial markers (claudin-4, CD40, and AIRE) in thymoma and TSCC

We stained 53 cases of thymoma and TSCC with 3 medullary markers (Table 1).

**Fig. 2:**
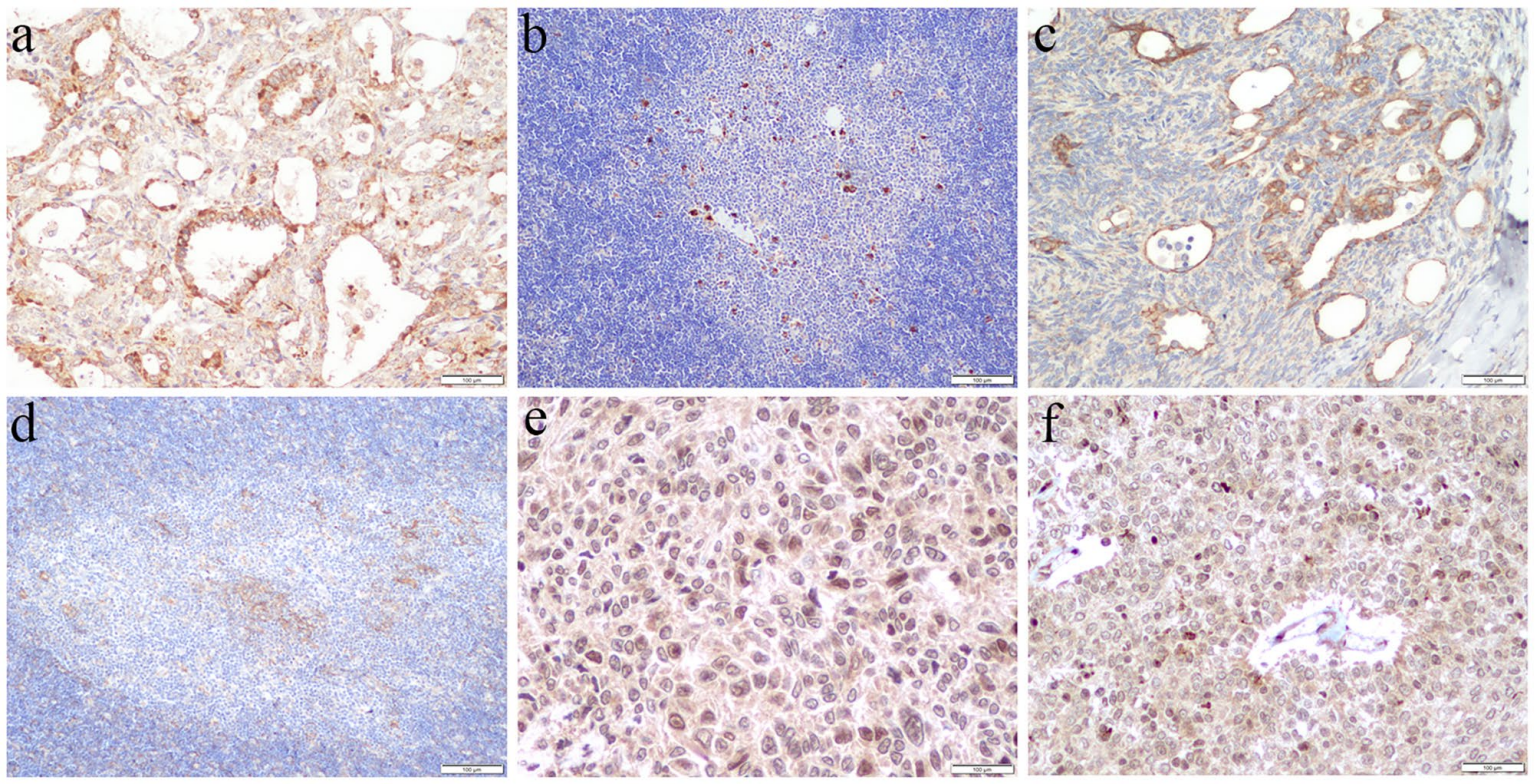
Expression of medullary epithelial markers in thymoma and TSCC. (a) CD40 in AB thymoma × 200; (b) CD40 in B1 thymoma × 100; (c) Claudin-4 in AB thymoma × 200; (d) Claudin-4 in B1 thymoma × 100; (e) AIRE in A thymoma × 400; (f) AIRE in AB thymoma × 400

CD40 is expressed in all types of thymoma and TSCC. A total of 60.0% (3/5) of type A, 61.5% (8/13) of type AB, 56.5% (13/23) of type B, 40.0% (2/5) of MNT, and 57.1% (4/7) of TSCC were CD40 positivity. For AB thymoma, CD40 was more strongly expressed in tumor cells around the cysts (Fig. 2A). In B1 thymoma, CD40 was more expressed in the medullary islands than in the cortical areas (Fig. 2B). Claudin-4 positive was observed in 20.0% (1/5) of type A, 69.2% (9/13) of type AB, 43.5% (10/23) of type B, 40.0% (2/5) of MNT, 85.7% (6/7) of TSCC. In AB thymoma, claudin-4 was more strongly expressed in the epithelial cells surrounding the cysts in the A component (Fig. 2C). Similarly, claudin-4 expression was stronger in medullary islands of B1 thymoma (Fig. 2D). The rate of AIRE expression was 100.0% (5/5) in type A (Fig. 2E), 76.9% (10/13) in type AB (Fig. 2F), 47.8% (11/23) in type B, 40.0% (2/5) in MNT, 28.6% (2/7) in TSCC. Table 2 summarized the expression of medullary epithelial markers in the differential diagnosis. The expression of AIRE was higher in type A and AB than in B3 thymoma (p < 0.05). No significant differences in the rest groups (p > 0.05).

### Correlation between thymic epithelial markers expression and histological classification

**Fig. 3:**
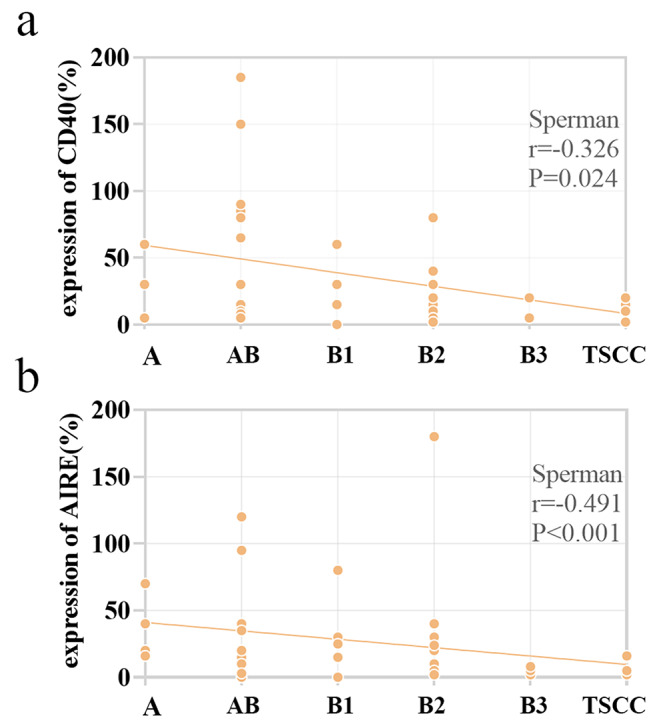
Associations between histological classification and CD40 and AIRE expression

The score of thymic markers expression was used to assess the associations between thymic epithelial markers expression and histological classification. With histological classification moving from A to AB, B1, B2, B3, and TSCC, we found an inverse correlation between thymoma histological classification and CD40 and AIRE expression (Spermanr= -0.326, p = 0.024; Spermanr= -0.419, p < 0.001 respectively) (Fig. 3). There was no correlation between thymoma histological classification and β5t, PRSS16, cathepsin V and claudin-4 expression (p > 0.05).

**Table 3:** Corticomedullary epithelial immunophenotype of thymomas and TSCC

Classification	Corticomedullary epithelial immunophenotype (%)				P vs. type B
	Cortical	Medullary	Bidirectional	Not shown	
MNT	0	4(80.0)	0	1(20.0)	0.009
A	0	4(80.0)	1(20.0)	0	0.009
AB	5(38.5)	5(38.5)	3(23.1)	0	0.194
B1	1(20.0)	1(20.0)	2(40.0)	1(20.0)	0.378(vs. B2)
B2	7(53.8)	1(7.7)	5(38.5)	0	1.000(vs. B3)
B3	4(80.0)	1(20.0)	0	0	1.000(vs. B1)
TSCC	1(14.3)	4(57.1)	2(28.6)	0	0.031

### Corticomedullary epithelial immunophenotype in 53 cases of thymoma and TSCC

**Fig. 4:**
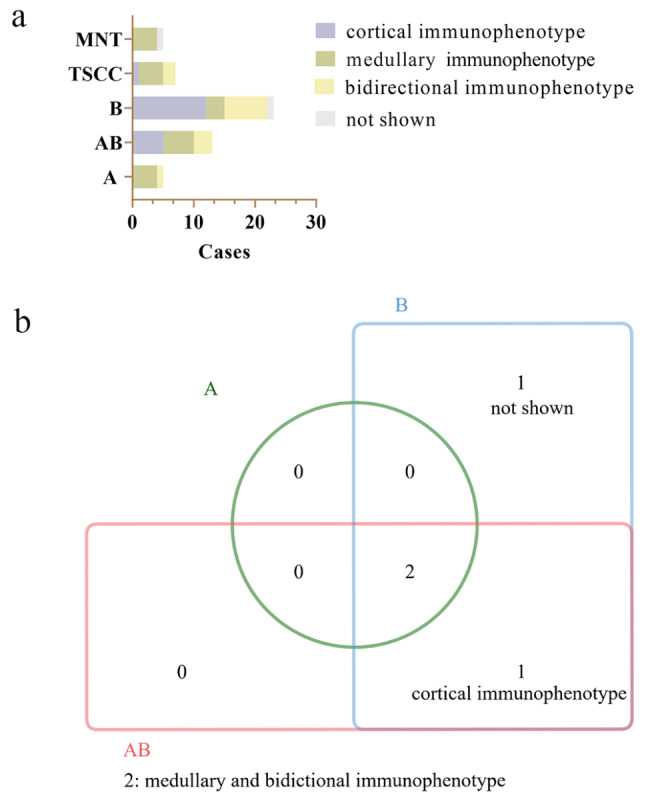
Comparison of corticomedullary epithelial immunophenotype in thymomas and TSCC

The comparison of corticomedullary epithelial immunophenotype in thymomas and TSCC is shown in Fig. 4. The results are summarized in Table 3. A total number of 18 (34.0%) patients with a cortical epithelial immunophenotype, 20 (37.7%) patients with a medullary epithelial immunophenotype, 13 (24.5%) patients with a bidirectional epithelial immunophenotype, and 2 (3.8%) patients with null of corticomedullary epithelial immunophenotype.

Cortical epithelial immunophenotype was found in 52.2% (12/23) of B thymoma, 38.5% (5/13) of AB thymoma, 14.3% (1/7) of TSCC, and not found in MNT and A thymoma. In B thymoma, the proportion of cases with a cortical epithelial immunophenotype increases successively in B1, B2, and B3. All types of thymoma and TSCC showed medullary epithelial immunophenotype. Medullary epithelial immunophenotype was found in 80.0% (4/5) of type A, 13.0% (3/23) of type B, 38.5% (5/13) of type AB, 80.0% (4/5) of MNT, 57.1% (4/7) of TSCC. Corticomedullary bidirectional epithelial immunophenotype was observed in 23.1% (3/13) of type AB, 40.0% (2/5) of B1 thymoma, 38.5% (5/13) of B2 thymoma, 28.6% (2/7) of TSCC, 20.0% (1/5) of A thymoma and not found in MNT, and B3 thymoma. Compared with type B, the portion of the medullary epithelial immunophenotype of MNT, A thymoma, and TSCC was significantly higher (p < 0.05). No correlation was found between type B and AB in corticomedullary epithelial immunophenotype (p > 0.05).

### The relationship between thymic epithelial markers expression and survival

**Fig. 5:**
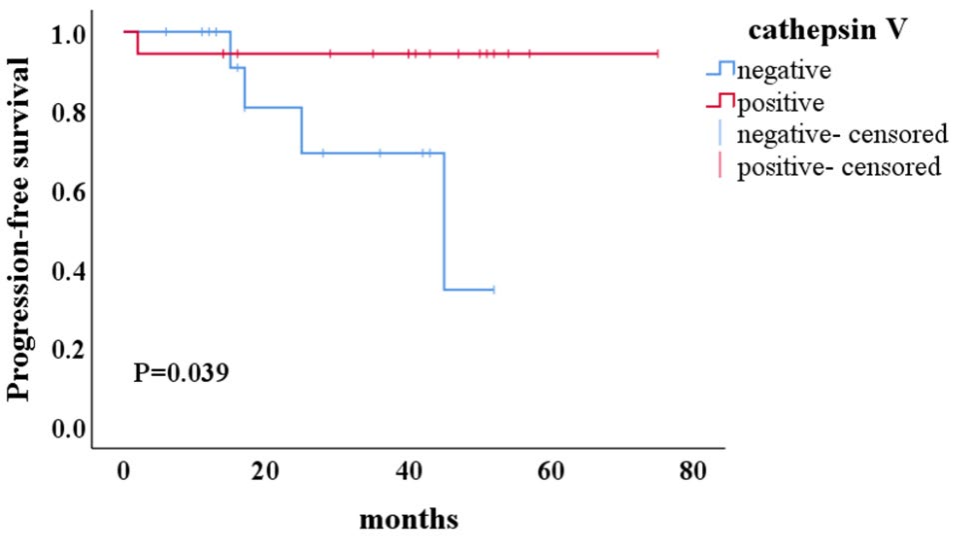
Kaplan-Meier curves of cathepsin V expression and PFS in thymomas and TSCC

Associations between thymic epithelial markers expression, Masaoka-Koga stage, WHO classification, and survival status are summarized in Table 4. Cathepsin V was significantly correlated with PFS (p < 0.05). High cathepsin V expression was associated with a good prognosis and a longer PFS (Fig. 5), but not for OS. B2, B3, and TSCC have worse PFS and OS than type A, AB, B1, and MNT (p < 0.05). Although no relationship was found between PFS and PRSS16, claudin-4 expression, there was a trend toward improved PFS (p = 0.078, p = 0.069, respectively) with PRSS16 positive and claudin-4 negative. There were no significant associations between PFS, OS, and Masaoka-Koga stage, β5t, CD40, and AIRE expression (p > 0.05). The small number of death and recurrence precluded multivariate analysis.

**Table 4:** Univariate analysis of PFS and OS according to clinicopathologic patterns and thymic epithelial markers expression

parameters		PFS		OS	
		Median PFS (months)	P	Median OS (months)	P
β5t	+	51.327	0.612	51.527	0.722
	-	61.494		70.000	
PRSS16	+	70.706	0.078	70.941	0.413
	-	41.240		57.275	
Cathepsin V	+	70.944	0.039	71.167	0.329
	-	39.560		56.111	
CD40	+	67.211	0.437	67.421	0.700
	-	47.056		62.455	
Claudin-4	+	42.532	0.069	58.266	0.352
	-	71.133		71.133	
AIRE	+	50.582	0.294	63.769	0.323
	-	58.829		63.744	
WHO subtype	A, AB, MNT, B1	—	0.006	—	0.040
	B2, B3, TSCC	—		—	
Masaoka-Koga stage	I	70.438	0.296	70.688	0.686
	II, III, IV	47.451		59.923	

PFS: Progression-free survival; OS: Overall survival

### The relationship between thymic epithelial markers expression and Masaoka-Koga stage

Excluding biopsy specimens, a total of 52 thymomas and TSCC were studied, including 21 stage I, 17 stage II, 13 stage III, and 1 stage IV, and the association between Masaoka-Koga stage and immunohistochemical expression is summarized in Table 5. There was a significant difference in the β5t and CD40 expression between Masaoka-Koga I and Masaoka-Koga II, III, IV. There was no significant difference in the PRSS16, cathepsin V, claudin-4, and AIRE expression between Masaoka-Koga I and Masaoka-Koga II, III, IV.

**Table 5:** Masaoka-Koga stage of thymoma and TSCC according to thymic cortical and medullary markers expression

markers	Masaoka-Koga stage				P^a^
	I	II	III	IV	
β5t					
+	15(55.6)	7(25.9)	4(14.8)	1(3.7)	0.020
-	6(24.0)	10(40.0)	9(36.0)	0	
PRSS16					
+	11(39.3)	12(42.9)	4(14.3)	1(3.6)	0.862
-	10(41.7)	5(20.8)	9(37.5)	0	
Cathepsin V					
+	11(37.9)	11(37.9)	6(20.7)	1(3.4)	0.686
-	10(43.5)	6(26.1)	7(30.4)	0	
CD40					
+	16(53.3)	7(23.3)	7(23.3)	0	0.026
-	5(22.7)	10(45.5)	6(27.3)	1(4.5)	
Claudin-4					
+	11(39.3)	8(28.6)	9(32.1)	0	0.862
-	10(41.7)	9(37.5)	4(16.7)	1(4.2)	
AIRE					
+	14(48.3)	8(27.6)	7(24.1)	0	0.193
-	7(30.4)	9(39.1)	6(26.1)	1(4.3)	

^a^I vs. II, III, IV

## Discussion

In this study, we analyzed comprehensively the expression of thymic epithelial markers in thymoma and TSCC to examine whether they could serve as new markers for diagnosis, staging, and prognosis. We found that thymomas and TSCC showed evidence of both cortical and medullary differentiation. Most of the A thymomas, TSCC, and MNT showed a medullary epithelial immunophenotype. Type B thymomas primarily expressed a cortical epithelial immunophenotype, and B2 and B3 thymomas showed higher expression of cortical markers than B1. Type AB thymoma showed cortical or medullary epithelial immunophenotypes, and a similar proportion of cases also showed mixtures of cortical and medullary immunophenotypes.

Previous studies have discussed the cortical or medullary phenotype of thymoma, but the results are complex [4, 29, 30]. Some studies demonstrated the medullary notion of A thymoma and the cortical notion of B thymoma [4, 30], while other studies documented that medullary markers were mostly negative in A thymoma [29]. The sheets of tumor cells in MNT contain the features of A thymoma, and the similar expression of the mTEC marker suggested a common histogenesis of medullary origin [30, 31]. By morphology, type AB thymomas were composed of type A lymphocyte-poor components and type B-like lymphocyte-rich components. The corticomedullary differentiation of AB thymoma was unique showing mixed expression of cortical and medullary markers which were different from type A and B thymoma [29]. Our results were consistent with previous study indicating that AB thymoma is a distinct type of thymoma rather than a mixture of type A and type B thymoma [32].

In the differential diagnosis, in addition to applying CD20, CD3, CD5 and other immunological markers to identify background lymphocytes, β5t, PRSS16, and cathepsin V can also be applied for diagnosis. Cathepsin V is mainly expressed in type B and AB thymomas, with less expression in A thymomas. The combined application of cathepsin V and cathepsin S helped identify TSCC and B3 thymomas [22]. The expression of β5t was like that of cathepsin V and was mainly expressed in type B and AB thymomas, compared with type A thymomas and TSCC, and its expression helped to identify TSSC and B3 thymomas [18, 19]. Eriko S et al. [33] examined AIRE mRNA in 45 thymomas and found that AIRE mRNA levels were higher in B2 thymoma than in other types of thymomas, AIRE mRNA expression was lowest in AB thymoma. Some studies reported that MNT could mix with type A, AB, and B thymoma [34]. Our study proved that cortical epithelial markers (β5t, PRSS16, and cathepsin V) could distinguish AB and B thymomas from MNT and that the positive rate of cortical epithelial markers was higher in AB and B thymomas, especially in biopsy specimens with abundant lymphocyte infiltration around epithelial nodules. Moreover, our results indicated that A and AB thymomas could compose of spindle cells, the application of β5t and PRSS16 helped to identify type AB and A thymomas. It is important to identify type A and B3 thymomas because of the significant difference in disease-free and overall survival [15]. In this study, we found that the medullary epithelial marker AIRE was found to help differentiate B3 from A thymomas. In general, the perivascular spaces are obvious in B3, and the tumor cells are large, polygonal, and slightly or moderately atypical, most of them can be recognized from A thymomas, but a few B3 thymomas can show spindle cell features, which are not easily distinguished from A thymomas. Very few atypical A thymomas, with increased cell density with heterogeneity, necrosis, and mitotic, are also easily confused with B3 thymomas. In our results, the expression rate of AIRE in type A thymoma was 100%, while in B3 thymoma it was completely negative. Combined with histologic morphology, the application of AIRE is helpful for differential diagnosis. AIRE is mainly expressed in mTECs and plays a crucial role in promoting self-tolerance [35]. Thymoma has an association with autoimmune disease, and myasthenia gravis (MG) was the commonest, which usually occurs in type B thymomas, and the risk of MG increased from A to B3 thymoma [36]. The level of AIRE mRNA in type A, AB, and B1 thymoma was significantly higher than that in the B2, B3, and C (thymic carcinoma) thymoma [37]. AIRE expression in thymoma with the presence of MG was significantly lower than that in the simple thymoma without autoimmune disease [37]. Our results suggest that the AIRE expression declined from A, AB, B1, B2, B3, and TSCC, which is similar to previous studies and indicates that AIRE may be associated with the presence of MG in thymoma.

Staging, histological classification, and surgical resection status of thymoma are important factors in prognosis. In 2020, a Meta-analysis study found that the expression of many markers was associated with higher Masaoka stage of thymic tumors, including EGFR, Glut-1, EMA, Bcl-2, etc. [38]. CD40 is expressed in many tumors, but its significance is different. CD40 expression in breast and gastric cancers was associated with an earlier stage [39, 40], whereas in esophageal squamous cell carcinoma CD40 expression was associated with a more advanced pathological stage, poorer differentiation, and higher rates of lymph node metastasis, predicting an increased potential for tumor progression and metastasis [41]. In the present study, β5t and CD40 expression correlated with low Masaoka-Koga staging. In the National Comprehensive Cancer Network (NCCN) guidelines of thymoma and thymic carcinoma (Version 1.2023 — December 15, 2022), postoperative management was guided by Masaoka-Koga stage especially radiation therapy [42]. Our result indicates that β5t and CD40 expression may help with postoperative treatment. We also evaluated the TNM stage by using the 8th edition AJCC/UICC staging manuals. As lymph node dissection was only performed in a few cases in our center and the rarity of distant metastasis, we only assessed the pT stages. There was no significant difference in the expression of thymic epithelial markers between different pT stages.

Cathepsin V is involved in the prognosis of many tumors. High expression of cathepsin V in ductal carcinoma in situ correlates with poor prognostic factors (histological grading, hormone receptor negativity, and HER-2 positivity) [43]. Both cathepsin V and mRNA expression were higher in hepatocellular carcinoma than in normal liver tissue, and immunohistochemical results showed that high cathepsin V expression was associated with shorter OS and DFS [44]. Kiuchi S et al. found that the expression patterns of cathepsin V and cathepsin S helped identify TSCC and B3 thymomas and the recurrence rate was higher in cathepsin V-negative patients than in cathepsin V-positive patients with TSCC [22]. In the present study, cathepsin V expression was associated with prolonged PFS. This observation was similar to those of Kiuchi S et al. [22]. However, our study had some limitations. The sample size is limited, so further sample size expansion is needed to make thymic epithelial markers can be applied more reliably.

## Conclusion

In conclusion, our findings, for the first time, estimated the expression of thymic cortical (β5t, PRSS16, and cathepsin V) and medullary epithelial markers (AIRE, CD40, and claudin-4) in differential diagnosis, Masaoka-Koga stage, histological classification, and prognosis of thymic tumors. Our results revealed that the potential differential diagnosis utility of β5t, PRSS16, cathepsin V, and AIRE. CD40 and β5t expression were associated with the Masaoka-Koga stage of thymoma and TSCC. Cathepsin V expression can facilitate the prognosis of patients. Moreover, our results support the morphological distinction of thymoma subtypes.

## Data Availability

The datasets used and/or analyzed during the current study are available from the corresponding author on reasonable request.

## Abbreviations

TSCC: Thymic squamous cell carcinomas
MNT: Micronodular thymoma with lymphoid stroma
TETs: Thymic epithelial tumors
WHO: World Health Organization
cTECs: Cortical thymic epithelial cells
mTECs: Medullary thymic epithelial cells
OS: Overall survival
PFS: Progression-free survival
MG: myasthenia gravis
β5t: thymoproteasome-specific subunit β5t
PRSS16: Thymus-specific serine protease 16
AIRE: Autoimmune regulator
PBS: Phosphate-buffered saline
